# Spatial Distribution and Post-COVID-19 Health Complications in Older People: A Brazilian Cohort Study

**DOI:** 10.3390/jcm14134775

**Published:** 2025-07-06

**Authors:** Franciele Aline Machado de Brito, Carlos Laranjeira, Stéfane Lele Rossoni, Amira Mohammed Ali, Maria Aparecida Salci, Lígia Carreira

**Affiliations:** 1 Department of Postgraduate Nursing, State University of Maringá, Avenida Colombo, 5790-Campus Universitário, Maringá 87020-900, Brazil; pg54936@uem.br (F.A.M.d.B.); masalci@uem.br (M.A.S.); lcarreira@uem.br (L.C.); 2 School of Health Sciences, Polytechnic University of Leiria, Campus 2, Morro do Lena, Alto do Vieiro, Apartado 4137, 2411-901 Leiria, Portugal; 3 Centre for Innovative Care and Health Technology (ciTechCare), Polytechnic University of Leiria, Campus 5, Rua das Olhalvas, 2414-016 Leiria, Portugal; 4 Comprehensive Health Research Centre (CHRC), University of Évora, 7000-801 Évora, Portugal; 5 Postgraduate Department of Health Sciences, State University of Maringá, Avenida Colombo, 5790-Campus Universitário, Maringá 87020-900, Brazil; pg56050@uem.br; 6 Department of Psychiatric Nursing and Mental Health, Faculty of Nursing, Alexandria University, Smouha, Alexandria 21527, Egypt; amiramali@alexu.edu.eg

**Keywords:** COVID-19, post-acute COVID-19 syndrome, chronic disease, older people, Brazil

## Abstract

**Background/Objectives:** In the aftermath of the COVID-19 pandemic, individuals infected with SARS-CoV-2 have progressively displayed a range of symptoms linked to protracted COVID during the post-acute phase of illness. Concurrently, in several nations globally, the phenomenon of population aging has been intensifying. In this scenario, the aged population has become both vulnerable and high-risk during the acute phase of COVID-19, and faces significant dangers associated with long-COVID. This study seeks to analyze the incidence and spatial distribution of health complications in older people affected by COVID-19, in the first year of the pandemic (2020), in the State of Paraná, as well as to identify the factors associated with the development of cardiovascular, neurological, respiratory, and metabolic diseases. **Method:** An observational and retrospective study was carried out in the Brazilian state of Paraná. Participants were randomly selected from two databases. A total of 893 older people (≥60 years) participated in the study 12 months after acute COVID-19 infection. Telephone questionnaires were applied between March and December 2021. The Moran index test, logistic regression, and Poisson models were used to analyze the data. **Results:** In terms of age, most participants (66%) were between 60 and 69 years old, 25.8% were between 70 and 79 years old, and 8.2% were 80 years old or older. Most participants were female (51.2%), white (98.1%), had a partner (69.8%), and had been hospitalized due to COVID-19 (59.3%). Cardiovascular diseases were the most frequent in the population (39.5%), followed by metabolic diseases (27.3%). The long-term use of medication was associated with the development of metabolic diseases (aOR = 9.8), cardiovascular diseases (aOR = 6.6), and diseases in multiple organic systems (aOR = 3.2); living alone was associated with neurological diseases (aOR = 2.5), and the age group of 80 years or older (aOR = 2.4) was associated with cardiovascular events. The spatial distribution showed that complications in body groups are distributed randomly among the health regions of the state, with no influence from neighboring locations. **Conclusions**: Post-COVID-19 health complications are more frequent in older adults who have comorbidities and long-term medication use. Therefore, long-term monitoring of these individuals and investment in public policies for rehabilitation and prevention of complications are necessary.

## 1. Introduction

The SARS-CoV-2 coronavirus has infected approximately 777 million individuals worldwide, resulting in 7 million deaths. Although COVID-19 is now fairly well studied, the pathophysiological mechanisms of the coronavirus still need to be better elucidated. The disease can aggravate pre-existing health conditions, as well as favor the occurrence of new complications after the acute phase of the disease [1,2].

In Brazil, almost 40 million cases and 714,534 deaths had been recorded by early 2025 [3]. It is estimated that, among survivors, 54% of those hospitalized and 34% of those not hospitalized have persistent symptoms [4], a condition known in the literature as long-COVID, which, according to the definition of the National Academy of Sciences, Engineering and Medicine, are symptoms that last for three months or more, after the acute phase of the infection, and that can affect one or multiple organic systems at the same time [3,4,5,6]. Previous research has identified numerous symptoms, illnesses, or complications that can arise after COVID-19, in particular cardiovascular, respiratory, neurological, and metabolic diseases [7,8]. Immune dysregulation, autoimmunity, endothelial abnormalities, and coagulation disorders are believed to account for a large proportion of these events [9,10,11].

With the increase in life expectancy and the consequent aging of the population, the incidence of chronic diseases—such as Arterial Hypertension, Diabetes Mellitus, and Dyslipidemia—that worsen the health of older adults has increased. In addition, several risk factors contribute to the incidence of these conditions, such as a sedentary lifestyle, excess weight, the presence of morbidities, and the long-term use of medication [12,13].

Although older persons represent a significant segment of those afflicted with severe COVID-19, less information exists regarding the incidence of health complications and risk factors associated with symptomatic long-COVID in this demographic. In most cohorts studying long-COVID, the average age of participants was under 60 years [14,15].

Older people, as a risk group, and even before the introduction of vaccines, suffered disproportionately from the effects of SARS-CoV-2 infection, presenting more severe clinical conditions [16]. Studies show that, among survivors, many presented sequelae and a greater susceptibility to acute events in the post-COVID period [17,18]. In this sense, studies with longitudinal designs are essential to observe the incidence of diseases or health complications in older people, given the vulnerability of this age group to SARS-CoV-2 infection. Likewise, it is necessary to identify the factors that may be associated with the development of these complications after the acute phase, since the pandemic generated multiple changes in the population’s lifestyle and behavior [12,19].

Therefore, this study aims to (a) analyze the incidence and spatial distribution of health complications in older people affected by COVID-19, in the first year of the pandemic (2020), in the State of Paraná; and, (b) identify the factors associated with the development of cardiovascular, neurological, respiratory, and metabolic diseases.

## 2. Materials and Methods

### 2.1. Study Design

This is an observational and follow-up study integrated into a larger project called “Longitudinal monitoring of adults and older people discharged from hospital due to COVID-19”, carried out by the State University of Maringá (UEM), in collaboration with the Health Department of the State of Paraná (SESA), Federal University of Pelotas (UFPEL), and Duke University, in the United States [20].

The study followed the guidelines of the Strengthening the Reporting of Observational Studies in Epidemiology (STROBE) checklist [21].

### 2.2. Sample and Recruitment

The study site was the State of Paraná, located in the Southern Region of Brazil, with 399 municipalities, divided into 22 health regions, grouped into four health macro-regions. The Human Development Index (HDI) is 0.769, ranking seventh in comparison with other states. Paraná is the fourth largest economy in the country and the first in this region [22].

In the last census of 2022, the state of Paraná had a population of 11,444,380 inhabitants. It was the fourth state with the highest number of cases and deaths from SARS-CoV-2 [3,22]. Study participants were randomly selected from the databases of the Influenza Epidemiological Surveillance Information System (SIVEP-Gripe) and Notifica COVID. When there was duplication of data between the two databases, information from SIVEP-Gripe was considered. For the selection of participants, the following inclusion criteria were defined: (a) older people (in Brazil, individuals aged 60 years or older are officially considered older people, in accordance with national legislation and public health standards [23]); (b) residents of the State of Paraná, with a positive result for COVID-19, with the RT-PCR test, between the months of March and December 2020. The study period encompasses individuals whose symptoms began prior to 7 June 2021, at which point over 75% of infections were with the alpha variant [24]. Individuals who had died were excluded.

Sampling followed a stratified approach to reduce selection and proportionality biases. The health macro-region of the participant’s residence (North, Northwest, East, and West) was considered, as well as the month in which the participant was notified of the disease infection (March to December 2020) or in which they were discharged from health services [20].

**Figure 1:**
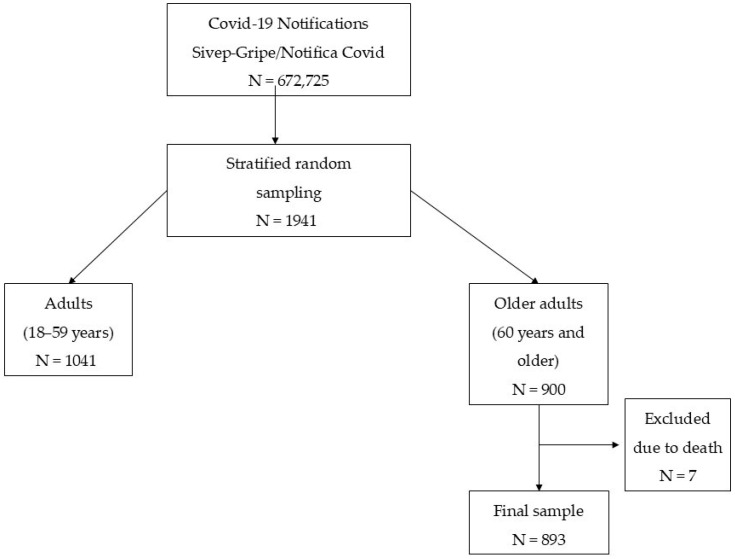
Flowchart illustrating the process of sample selection.

Of the 900 eligible participants, only 893 cases presented complete information and were analyzed. Figure 1 illustrates the sample selection process, from the initial identification in the databases to the final study sample.

### 2.3. Outcomes

Outcome variables were defined as those indicative of health complications, distributed across specific body organ systems. The group of cardiovascular diseases included: thrombosis (considered as any self-reported thrombotic event following COVID-19, regardless of anatomical location); cardiac arrhythmia; systemic arterial hypertension; acute myocardial infarction; angor pectoris; atherosclerosis; stroke; and cardiopulmonary embolism. Less common cardiac events not encompassed by the major categories were grouped under heart disease for interpretive clarity. The group of metabolic diseases included diabetes mellitus, thyroidopathy, dyslipidemia (defined as a self-reported diagnosis of abnormal lipid levels, as identified by a healthcare professional, in line with standard clinical thresholds), and obesity. In case of neurological diseases, the following were considered: Alzheimer’s disease, chronic neurological disease, epilepsy, and Parkinson’s. Finally, the group of respiratory diseases included pneumonia, asthma, bronchiectasis, bronchitis, chronic obstructive pulmonary disease (COPD), and pneumoconiosis.

All variables were scored dichotomously, indicating the presence or absence of the disease in the individual. Each systemic group was also classified dichotomously with the value 1 for the presence of at least one of the respective diseases, and 0 in the absence of all diseases.

### 2.4. Independent Variables

The independent variables related to the outcome included: sex (male or female); age range (60 to 69; 70 to 79; and 80 years or older); race (white; non-white); marital status (with partner; without partner); education (up to 8 years of study; 8 years of study or more); lives alone/with someone (yes, no); health macro-region (east; west; north; northwest); work situation up to 3 months before becoming infected with COVID-19 (yes, no); practicing physical activity before and after contracting COVID-19 (yes, no); tobacco use (yes, I currently smoke; no, I have never smoked; no, but I am an ex-smoker); long-term use of medication prior to COVID-19 infection (yes, no); need for hospitalization in the first occurrence of COVID-19 (yes, no); acute COVID-19 treatment location (outpatient; medical ward; Intensive Care Unit (ICU)); required ventilatory support during COVID-19 treatment (yes, invasive; yes, non-invasive; no); and body mass index >30 [indicating obesity] (yes; no).

### 2.5. Follow-Up Interview After 12 Months

Initial data collection was carried out with the SIVEP-Gripe and Notifica COVID databases and included the following variables: sex, age group, month of COVID-19 notification, place of disease treatment, municipality of residence, health region, health macro-region, and telephone contact. Twelve months after notification of acute COVID-19 infection, researchers established an initial telephone contact with participants in order to present the objectives of the study and request informed consent. If they agreed to participate in the study, a second telephone contact was planned according to their availability. Data collection took place from March to December 2021, and the interviewers were nurses who received training in interview facilitation skills (a total of 40 h) in order to mitigate interviewer bias. To this end, a structured form was developed and validated by the researchers, with thematic blocks on personal history, sociodemographic characterization, lifestyle habits, and history of COVID-19, in the acute and post-acute phases of the disease [20].

### 2.6. Statistical Analysis

For the descriptive analysis of the data, absolute and relative frequencies were used. For spatial analysis, a variable was created related to the number of body groups presenting diseases, which could vary from 0 to 4 per individual (0: none and 4: presented at least one symptom from each systemic group (cardiovascular, metabolic, neurological, and respiratory)). Subsequently, these data were grouped into the 22 health regions of Paraná based on the estimated population of older people in each region, in order to reduce proportional biases. The population data for each municipality were obtained from estimates of the resident population over 60 years of age in Brazilian municipalities in 2021 (contained in the IBGE) and subsequently grouped into each health region [18]. The quantities were weighted by the population and multiplied by 100,000, resulting in a variable that indicates how many systemic groups of sample participants were affected by COVID-19 for every 100,000 older people, in a given health region. To verify the existence of spatial dependence, the global Moran Index test was applied.

Furthermore, to verify the presence or absence of complications in each group of diseases, logistic regression models were adjusted, considering sociodemographic, health, and treatment variables as predictor variables, in order to identify factors associated with the presence of health complications. Thus, the regression models were applied for each outcome variable analyzed in the study; that is, there were four models, one for each organic system. The results presented correspond to the most parsimonious models, determined using the Stepwise selection method.

Finally, a Poisson regression model was also performed to assess which factors are associated with an increase in health complications. The response variable was the number of distinct body groups presenting health complications in each individual, which could vary from 0 to 4 (0: none and 4: presented at least one symptom from each systemic group (cardiovascular, metabolic, neurological, and respiratory)). To evaluate the adjusted regression models, the Shapiro–Wilk test was applied, which tested the normality of the residuals. After excluding all missing data, analyses were performed using the software R Core Team (version 4.3.2) with a significance level of 5%.

### 2.7. Ethics

The study was approved by the Research Ethics Committee of the State University of Maringá (reference: 4165272 and CAAE: 34787020.0.0000.0104, on 21 July 2020), in accordance with the National Health Council Resolution No. 466/2012. Regarding the data obtained from Notifica COVID, the authorization was granted by Hospital do Trabalho (reference: 4214589 and CAAE: 34787020.0.3001.5225, on 15 August 2020). All participants were apprised of the study’s aims and queried regarding their willingness to participate in the research. The consent terms were mailed to participants who consented, either through email or postal service, as per their preference.

## 3. Results

### 3.1. Sample Description

Table 1 presents the absolute and relative frequencies of the categorical variables under study. The sex distribution was balanced, with 51.2% women and 48.8% men. Regarding age group, 66% were between 60 and 69 years old, 25.8% were between 70 and 79 years old, and 8.2% were 80 years old or older. Regarding race, the majority (68.1%) declared themselves white, while the rest identified themselves as non-white (31.9%). Regarding marital status, 69.8% of the sample had a partner, and 84.1% did not live alone. Most participants (57.3%) had up to 8 years of education. The geographic distribution shows that individuals were largely from the east (44.2%), followed by the west (21.2%), north (18%), and northwest (16.6%) regions of the state. Furthermore, 36.5% of older people reported working up to three months before being infected with COVID-19 (Table 1).

The participants’ lifestyle habits revealed that 51.6% practiced physical activity before COVID-19, but only 38.1% continued with this practice after the infection. Regarding tobacco use, 3.3% declared themselves to be smokers, 21.2% were former smokers, and 75.5% had never smoked. Most participants (80.1%) used long-term medication before COVID-19 infection, and 36.8% had a body mass index (BMI) greater than 30 (Table 1). More than half of the sample (59.3%) reported having been hospitalized due to COVID-19. Among the treatment locations, 41.7% received care in the outpatient setting, 31.7% in the medical ward, and 26.6% required ICU care. Finally, 9.8% required invasive ventilatory support (Table 1).

Table 2 indicated that 31.6% of the participants had systemic arterial hypertension, followed by diabetes mellitus (18.8%), thyroid disease (7.4%), dyslipidemia (7.3%), and heart disease (5.7%). On the other hand, there were no records of epilepsy and pneumoconiosis. Regarding the affected organic systems, 39.5% of participants reported at least one cardiovascular disease, followed by those with metabolic diseases (27.3%), neurological diseases (2.9%) and finally, respiratory diseases (3.7%).

### 3.2. Spatial Analysis

**Figure 2:**
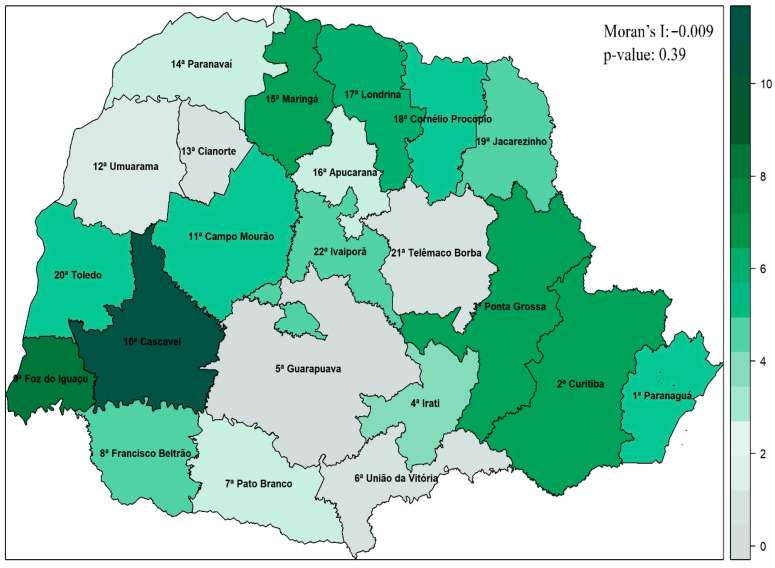
Distribution of health complications resulting from COVID-19 in older people in the state of Paraná, weighted by the estimate of the older population multiplied by 100,000.

The map presented in Figure 2 shows the distribution of the proportion of body systems affected by COVID-19 in older people for each health region, weighted by the region’s estimated population aged 60 or over and multiplied by 100,000. The Cascavel region stands out from the others, as the region presenting the most health complications in older people, proportionally to the local population. The Guarapuava region was the one with the fewest complications, followed by Telêmaco Borba and Cianorte. However, the calculated Moran’s Index indicated that there is no significant spatial dependence (I = −0.009; *p*-value = 0.39), that is, the proximity between regions does not affect the level of health complications in neighboring locations.

### 3.3. Regression Analysis

For the outcome variable “cardiovascular diseases”, and based on the model estimates, the age group and long-term use of medication were significant, indicating that individuals over 80 years old were 2.4 times more likely to develop cardiovascular diseases, compared to those between 60 and 69 years old. Furthermore, older people who used long-term medications before COVID-19 had a 6.6% higher risk of developing these diseases, compared to those who did not use them (Table 3).

Table 3 also shows an association between sex and long-term use of medication with the occurrence of metabolic diseases. Males were 41% less likely to develop these conditions, compared to females. On the other hand, older people who used long-term medications before COVID-19 were 9.8 times more likely to have metabolic diseases, when compared to those who did not use them.

As for neurological diseases, living alone proved to be a significant factor. Older people who lived alone had a 2.5 times greater risk of developing neurological changes, compared to those who lived with someone (Table 3). In the case of respiratory diseases, the model did not identify significance in any of the variables evaluated in the research.

Poisson regression was used to analyze the number of affected body systems. Model estimates showed significance for long-term use of medication before COVID-19. Older people who were taking medication before the infection were 3.2 times more likely to have an increase in the number of body systems affected (Table 4).

## 4. Discussion

The current study identified arterial hypertension, diabetes mellitus, and dyslipidemia as the most frequent complications in the population. The proportion of older people with low physical activity, high BMI, and long-term use of medication may be related to these diseases. The results also show that more than a third of the participants had at least one cardiovascular disease, followed by metabolic diseases. These findings corroborate with the available literature. In a study analyzing the period before the pandemic (2006 to 2019) and the years 2020 to February 2022, the practice of physical activity decreased during the pandemic, thus increasing sedentary behavior. Diseases such as high blood pressure, diabetes mellitus, and obesity also increased during the pandemic period, leading to an increase in chronic diseases, which are considered risk factors for various complications and death [19]. Another study that evaluated a sample of 162,673 older people found that Diabetes Mellitus showed an increasing trend between 2006 and 2021, as did overweight and obesity, which increased significantly. The pandemic caused changes in lifestyle and harmful behaviors among the population [12]. However, increased life expectancy and aging are factors that contribute to the incidence of chronic non-communicable diseases. In addition, the pandemic had other effects, such as delays in the diagnosis and treatment of diseases not related to COVID-19, worsening of mental illness, and economic impacts such as increased unemployment, decreased family income, and food insecurity [12,13,17].

Regarding factors associated with the development of cardiovascular, metabolic, respiratory, and neurological diseases, few studies have monitored individuals infected with COVID-19, observing the incidence of these complications. Most research has analyzed risk factors for persistent COVID-19 symptoms [25]. Cardiovascular diseases are most prevalent in the age group ≥80 years and in participants with long-term use of medications. Although a greater vulnerability to cardiovascular complications in individuals aged 80 years and older is clinically expected, the empirical confirmation and quantification of this association in a post-COVID-19 context adds epidemiological value and reinforces the importance of targeted surveillance in this age group. In a cohort involving 153,760 individuals who tested positive for COVID-19 and were followed for 12 months, the incidence rates of cardiovascular complications after the acute phase of the disease were significantly higher than those observed pre-exposure to SARS-CoV-2 [18]. This cohort also revealed that the risk of dysrhythmias, pericarditis, myocarditis, angina, acute coronary disease, heart failure and cardiogenic shock is higher when affected individuals are compared to the control group without COVID-19, and these occurrences are independent of other cardiovascular risk factors, such as obesity, chronic kidney disease or hyperlipidemia [18].

Studies show that, as age advances, changes related to the cardiovascular system may arise, especially after infection by SARS-CoV-2. In a recent systematic review, advanced age and the number of comorbid conditions were considered risk factors for long-COVID and health complications [17]. The same review highlighted that COVID-19 can cause coagulation disorders, which increase thromboembolic events such as thrombosis, heart disease, acute myocardial infarction, stroke, and pulmonary embolism, as some of the participants in this study presented. Furthermore, comorbidities such as high blood pressure and diabetes mellitus, prevalent in older adults, alter microvascular function, potentially compromising other organic systems [17].

Regarding the development of metabolic diseases, female gender and the long-term use of medications are related to changes in this system. Previous studies indicate that the main risk factors for chronic diseases are systolic blood pressure, BMI, high blood glucose and cholesterol levels, and an inadequate diet. Such factors result in morbidities and, consequently, in the long-term use of medications [19,26].

Considering that cardiometabolic diseases are among the main comorbidities associated with COVID-19 complications and mortality, public health measures aimed at reducing the burden of chronic conditions—such as regulating the consumption of ultra-processed foods and sugary beverages—may indirectly contribute to better outcomes in future public health emergencies [19,26].

Regarding the gender variable, research is unanimous regarding the association between long-COVID and the occurrence of post-COVID complications in females [27,28,29]. The physiological mechanisms by which women suffer more from the long-term effects of SARS-CoV-2 infection have not yet been fully elucidated; however, it is believed that hormonal changes, such as a drop in estrogen and immune responses with higher proportions of activated T cells (CD4 and CD8), are related to worse disease progression [28,29].

The findings obtained revealed that living alone appears to be associated with neurological diseases. Although there is no robust evidence to confirm this relationship, there are studies that argue that social isolation and loneliness can affect cognitive activity [30]. In this context, the COVID-19 pandemic was challenging for the older population due to the suffering generated by virus containment measures, which affected social relationships and caused increased levels of anxiety, depression, and loneliness [31,32]. Regarding the emergence of neurodegenerative diseases after infection, more studies are needed, with longer follow-ups, to build stronger evidence about this relationship [33].

The findings also reveal that long-term medication use is a predictor of increased chances of having a greater number of systemic groups affected. There is a consensus in the literature that COVID-19 can cause a multitude of symptoms, affecting one or multiple systems at the same time [34]. As previously discussed, the presence of morbidities and long-term use of medication associated with an organism recovering from COVID-19 can intensify physiological dysfunctions. The findings suggest that individuals with chronic conditions—typically treated with long-term medications—were more likely to develop post-COVID complications, likely due to the pre-existing physiological burden imposed by their underlying morbidities. Chronic diseases, for the most part, promote a state of persistent inflammation, in addition to micro and macrovascular damage, which compromises different body systems [2,35].

Presently, several challenges exist in the study and evaluation of long-COVID, encompassing complications associated with false-positive PCR tests and unreliable antigen/antibody tests that result in biases in COVID-19 case reporting, neglecting symptoms beyond the respiratory system and mild instances of long-COVID, as well as a limited comprehension of long-term symptoms subsequent to viral infections [8]. Consequently, it is imperative to enhance awareness of the risks associated with long-COVID, deliver training and education for healthcare professionals, instruct patients on self-assessment of their health conditions and long-COVID experiences, and augment investment in policies and funding for long-COVID research and management [8,36,37,38,39].

### Study Limitations

Some limitations of this study should be considered. First, the use of self-reported information obtained through interviews may be subject to recall bias on the part of participants. To mitigate this limitation, the data collection instrument was structured in thematic blocks in order to cover all relevant aspects of the investigation. Secondly, the study relied solely on self-reported data and lacked validation of the condition by the attending physician, as well as corroboration with electronic health records. The diagnosis of long COVID relied on self-reported symptoms and interview data, indicating that a more comprehensive collection of experiences might have reflected the real-world manifestation of long COVID more precisely. To enhance the accuracy and reliability of findings, future studies should use clinical assessments or medical records to precisely characterize long COVID symptoms and mitigate subjective bias. Thirdly, older individuals had a greater proportion of moderate and severe COVID-19 and, as a result, a higher rate of hospitalizations. This hinders definitive conclusions on whether variations are solely attributable to age or are confounded by illness severity. This can potentially constrain data analysis and restrict the generalizability of our findings. Further research should include a more extensive cohort of older outpatients during acute COVID-19. Fourthly, the sample analyzed may not adequately represent the panorama of the State of Paraná. However, a stratified approach was chosen to minimize possible selection and proportionality biases. Fifthly, dichotomizing certain variables may have resulted in the loss of important information, limiting the identification of more refined associations. Sixthly, the diseases or complications that affected participants after infection with SARS-CoV-2 may be related to genetic factors, lifestyle habits, or the senescence process itself. Despite the numerous variables being examined, additional variables, such as reinfections, the extent of COVID-19 vaccination coverage, or vaccine-induced immune responses, should be examined in subsequent research. All participants were initially diagnosed with COVID-19 before June 2021, when either the wild-type or alpha variant predominated, and before the extensive distribution of COVID-19 vaccines [24]. The presence of a control group composed of individuals with no history of COVID-19 will also allow a more accurate assessment of the incidence of these events. However, the databases consulted did not provide records of people without a diagnosis of the disease.

## 5. Conclusions

According to the results, the long-term use of medication before COVID-19 was shown to contribute to the risk of developing cardiovascular and metabolic diseases and the number of organ systems affected. Males were less likely to develop metabolic diseases, while living alone increased the chances of developing neurological diseases. The number of systemic groups affected in older people in Paraná is distributed randomly among the health regions of the state, without influence from neighboring locations. Additional prospective long-term follow-up studies investigating health conditions after SARS-CoV-2 infection are needed to monitor the complications of COVID-19 in the long term and to build stronger evidence and thus validate the results obtained. Further interventions designed to enhance recovery and welfare for this vulnerable population should be evaluated through comparative trials.

## Figures and Tables

**Table 1:** Sociodemographic characteristics, lifestyle habits, and history of COVID-19 (N = 893).

Variables	n (%)
**Sex**	
Male	436 (48.80)
Female	457 (51.20)
**Age range (years)**	
60 a 69	589 (66)
70 a 79	230 (25.80)
≥80	74 (8.20)
**Race**	
White	393 (68.10)
Non-white	184 (31.90)
**Marital status**	
With partner	481 (69.80)
Without partner	208 (30.20)
**Education (years)**	
<8	315 (57.30)
≥8	235 (42.70)
**Living alone**	
No	624 (84.10)
Yes	118 (15.90)
**Macroregional health**	
East	395 (44.20)
West	189 (21.20)
North	161 (18)
Northwest	148 (16.60)
**Work activity before COVID-19**	
No	409 (63.50)
Yes	235 (36.50)
**Physical activity before COVID-19**	
No	314 (48.40)
Yes	335 (51.60)
**Physical activity after COVID-19**	
No	402 (61.90)
Yes	247 (38.10)
**Tobacco use**	
Yes, smokes currently	22 (3.30)
No, never smoked	502 (75.50)
No, but I’m an ex-smoker	141 (21.20)
**BMI > 30 (obesity)**	
No	344 (63.20)
Yes	200 (36.80)
**Long-term use of medication before COVID-19**	
No	134 (19.90)
Yes	538 (80.10)
**Had to be hospitalized due to COVID-19**	
No	363 (40.70)
Yes	529 (59.30)
**COVID-19 treatment location**	
Outpatient (mild symptoms)	372 (41.70)
Medical ward (moderate symptoms)	283 (31.70)
Intensive Care Unit [ICU] (severe symptoms)	238 (26.60)
**Ventilatory Support**	
Non-invasive	644 (90.20)
Invasive	70 (9.80)

BMI, Body Mass Index; ICU, Intensive Care Unit.

**Table 2:** Presence or absence of disease and body system affected after COVID-19 (N = 893).

Variables	n (%)
**Cardiovascular diseases**	353 (39.50)
**Thrombosis**	
No	877 (98.20)
Yes	16 (1.80)
**Cardiac Arrhythmia**	
No	849 (95.10)
Yes	44 (4.90)
**Heart disease**	
No	842 (94.30)
Yes	51 (5.70)
**Systemic Arterial Hypertension**	
No	611 (68.40)
Yes	282 (31.60)
**Acute Myocardial Infarction**	
No	878 (98.30)
Yes	15 (1.70)
**Angor pectoris**	
No	886 (99.20)
Yes	7 (0.80)
**Atherosclerosis**	
No	883 (98.90)
Yes	10 (1.10)
**Stroke**	
No	872 (97.60)
Yes	21 (2.40)
**Cardiopulmonary embolism**	
No	864 (96.80)
Yes	29 (3.20)
**Metabolic Diseases**	244 (27.30)
**Diabetes Mellitus**	
No	725 (81.20)
Yes	168 (18.80)
**Thyroidopathy**	
No	827 (92.60)
Yes	66 (7.40)
**Dyslipidemia**	
No	828 (92.70)
Yes	65 (7.30)
**Obesity**	
No	879 (98.40)
Yes	14 (1.60)
**Neurological Diseases**	26 (2.90)
**Alzheimer Disease**	
No	878 (98.30)
Yes	15 (1.70)
**Chronic neurological disease**	
No	888 (99.40)
Yes	5 (0.60)
**Epilepsy**	
No	893 (100.00)
**Parkinson**	
No	882 (98.80)
Yes	11 (1.20)
**Respiratory Diseases**	33 (3.70)
**Pneumonia**	
No	877 (98.20)
Yes	16 (1.80)
**Asthma**	
No	886 (99.20)
Yes	7 (0.80)
**Bronchiectasis**	
No	891 (99.80)
Yes	2 (0.20)
**Bronchitis**	
No	886 (99.20)
Yes	7 (0.80)
**COPD**	
No	887 (99.30)
Yes	6 (0.70)
**Pneumoconiosis**	
No	893 (100.00)

**Table 3:** Logistic regression model estimates for cardiovascular, metabolic, and neurological disease outcome variables.

Variables	Estimate	Standard Error	*p*-Value	aOR	(95% CI)
**Cardiovascular Diseases**					
Age range: 70 to 79 years	−0.01	0.22	0.950	0.99	(0.64; 1.53)
Age range: ≥80 years	0.88	0.42	**0.036**	2.41	(1.09; 5.71)
Long-term use of medication before COVID-19—Yes	1.89	0.26	**<0.001**	6.63	(4.05; 11.21)
**Metabolic Diseases**					
Sex: Male	−0.52	0.19	**0.006**	0.59	(0.41; 0.86)
Long-term use of medication before COVID-19—Yes	2.28	0.36	**<0.001**	9.81	(5.06; 21.42)
**Neurological Diseases**					
Lives alone: Yes	0.92	0.43	**0.003**	2.52	(1.04; 5.78)

aOR: Adjusted Odds Ratio; CI: Confidence Interval. Bold means *p* < 0.05.

**Table 4:** Estimates of the Poisson regression model for the variable count of affected body systems.

Variables	Estimate	Standard Error	*p*-Value	aOR	(95% CI)
Sex: Male	−0.16	0.08	**0.056**	0.85	(0.72; 1)
Long-term use of medication before COVID-19—Yes	1.16	0.16	**<0.001**	3.20	(20.35; 4.48)

aOR: Adjusted Odds Ratio; CI: Confidence Interval. Bold means *p* < 0.05.

## Data Availability

The raw data supporting the conclusions of this article will be made available by the authors on request.
